# Parallel Single Cancer Cell Whole Genome Amplification Using Button-Valve Assisted Mixing in Nanoliter Chambers

**DOI:** 10.1371/journal.pone.0107958

**Published:** 2014-09-18

**Authors:** Yoonsun Yang, Joost F. Swennenhuis, Hoon Suk Rho, Séverine Le Gac, Leon W. M. M. Terstappen

**Affiliations:** 1 Medical Cell BioPhysics Group, MIRA Institute for Biomedical Technology and Technical Medicine, University of Twente, Enschede, The Netherlands; 2 Mesoscale Chemical Systems Group, MESA+ Institute for Nanotechnology, University of Twente, Enschede, The Netherlands; 3 BIOS Lab on a Chip Group, MESA+ Institute for Nanotechnology, University of Twente, Enschede, The Netherlands; The University of Hong Kong, China

## Abstract

The heterogeneity of tumor cells and their alteration during the course of the disease urges the need for real time characterization of individual tumor cells to improve the assessment of treatment options. New generations of therapies are frequently associated with specific genetic alterations driving the need to determine the genetic makeup of tumor cells. Here, we present a microfluidic device for parallel single cell whole genome amplification (pscWGA) to obtain enough copies of a single cell genome to probe for the presence of treatment targets and the frequency of its occurrence among the tumor cells. Individual cells were first captured and loaded into eight parallel amplification units. Next, cells were lysed on a chip and their DNA amplified through successive introduction of dedicated reagents while mixing actively with the help of integrated button-valves. The reaction chamber volume for scWGA 23.85 nl, and starting from 6–7 pg DNA contained in a single cell, around 8 ng of DNA was obtained after WGA, representing over 1000-fold amplification. The amplified products from individual breast cancer cells were collected from the device to either directly investigate the amplification of specific genes by qPCR or for re-amplification of the DNA to obtain sufficient material for whole genome sequencing. Our pscWGA device provides sufficient DNA from individual cells for their genetic characterization, and will undoubtedly allow for automated sample preparation for single cancer cell genomic characterization.

## Introduction

For the characterization of tumors, the expression of specific proteins or genes is usually provided as present or absent, for instance, ER+ or ER−, HER2+ or HER-, and EGFR mutation present or absent. This determination has important consequences for the immediate therapeutic decisions and to subject patients to specific therapies. For example, patients whose cancer cells have an amplification of the ERBB2 (Her2) gene are most likely to benefit from Her2 targeting drugs such as Trastuzumab. [1] Unfortunately, tumors are much more complex: expression levels can vary extensively within a tumor and are subject to change during the course of the disease. [2]–[4] For instance, somatic mutations can be present only in a small subset of the tumor[5] and tumor cells can become resistant to therapy associated with genetic alterations. To demonstrate the presence and extent of this heterogeneity in tumor cells analysis at the single cell level is required not to miss this important information [5]–[7].

To investigate the genome of a single cell in an extensive and reliable way, the whole genome must be amplified while maintaining the original representation of the genes to perform downstream analysis such as whole genome sequencing [6]–[8], array comparative genome hybridization (aCGH) [9], [10], or real-time quantitative PCR (RT-qPCR) [11], [12]. At this time all these techniques require tens of nano-grams to a few micro-grams of material of the whole genome. Multiple displacement amplification by phi 29 polymerase is one attractive approach for single cell whole genome amplification under isothermal conditions. [13] DNA amplification using the phi29 enzyme has the advantage that it can produce long DNA strands (>10 kb) in large quantities (up to 1∼2 µg) in a relatively short time (2 hours). [14]–[16] However, the extremely low concentration of DNA found in a single cell genome in the still large volume of the WGA mixture (20–50 µl) often gives rise to non-specific amplification and amplification biases. [17]–[19] In addition, sorting and manipulation of individual cells to perform single cell analysis can be very challenging and each manipulation can give rise to loss of material. Fluorescence activated cell sorting (FACS) [8], [20], micromanipulation [10], [11], laser capture microdissection (LCM) [9], [21] and DEP Array [22], [23] have all been applied for single cell isolation, but processing of the cells to obtain DNA for downstream analysis (e.g. lysis, nucleic acid isolation and amplification) has not or cannot be integrated. On the other hand, microfluidics and microfabricated structures allow for single cell manipulation while being easily coupled to a single cell analysis step. [24]–[26] Furthermore, microfluidics presents a key-advantage for WGA, since reactions take place in a much smaller volumes than when using traditional pipetting and microtubes (pico-liters versus micro-liters). This advantage has been particularly highlighted for WGA of single bacterial cells using reaction volumes of 60 nl resulting in a lower background and higher coverage with less amplification bias [27].

In microfluidic devices, mixing occurs naturally by passive diffusion. Especially the diffusion of large molecules such as phi29 polymerase takes longer than smaller molecules and limits the reliable and reaction speed in the microfluidic devices. Rotary mixer has been used to speed up this mixing process in microfluidic devices. [28], [29] However, the overall size of these structures limits the number of parallel reactors that can be placed on one device and the sample mixture must be transported to another reactor for the next step of the analysis. In addition, the surface area of a reactor is much larger, which may increase sample loss issues due to sticking at the channel walls. Alternatively, valve structures can be implemented in a continuous reactor for active mixing of the reagents.

Here, we introduce a microfluidic device for parallel single cell WGA. For demonstration a device with 8 parallel reaction chambers was designed and tested to load eight individual tumor cells that were subsequently lysed under alkaline conditions followed by the neutralization step and WGA. DNA from single cells were amplified using phi29 polymerase in the confined PDMS chamber for downstream off-chip analysis. Button valve-assisted mixing achieved WGA of individual tumor cells with a reagent volume of ∼20 nl as compared to 20 µl in traditional WGA reactions. *E.coli* DNA was first used as a model system to adapt WGA using phi29 polymerase, followed by individual cells from breast cancer cell lines for tumor scWGA. Quality of the on-chip amplified DNA was assessed by qPCR targeting 10 different genes.

## Experimental Methods

### Chip Fabrication and Device Preparation

The microfluidic devices were fabricated by multilayer soft lithography technique. [30], [31] First, the mask designs were prepared by CleWin software (WieWeb software, Hengelo, NL) and printed on a 5" soda lime glass by a mask generator LBPG Heidelberg DWL200 (Heidelberg Instruments Mikrotechnik GmbH). The master molds were fabricated by a photoresist-based photolithographic technique. Positive photoresist (AZ 40 XT, MicroChem Corp.) was spin-coated onto a 4" silicon wafer, and patterned using photolithography. For reliable operations of the microvalves, the cross-sectional shape of microchannels in the main fluidic channels was rounded by heating the mold at 140°C for one min after development. The top fluidic layer was produced by pouring uncured PDMS (GE RTV 615, elastomer: cross-linker = 5∶1) onto the mold to achieve a thickness of 7±0.5 mm. The bottom control layer was made by spin-coating uncured PDMS (elastomer: cross-linker = 20∶1) onto the master mold at 2500 rpm for 1 min. The resulting thickness of the control layer was 25±2 µm. The fluidic and control layers were cured for 45 min and 30 min, respectively, at 80°C. The fluidic layer was peeled off from the mold and holes for inlets and outlets were punched with a 25-gauge punch (Syneo Co., Angleton, TX, USA). Subsequently, the fluidic layer was aligned over the control layer using the alignment marks on the both layers under a stereomicroscope and the aligned layers were bonded by baking them at 80°C for 60 min. The bonded layers were then peeled off from the mold, and holes for control ports were punched. Finally, the multi-layer PDMS device was covered by a pre-cleaned glass slide (Fisher Scientific, Landsmeer, NL) and kept in the oven at 80°C for 12 h to enhance adhesion. Before use the devices were pre-coated by flowing a BSA solution (0.5 mg/ml) through the channels for 20 min. BSA solutions were pushed out by air.

### Whole Genome Amplification

Illustra GenomiPhi V2 DNA Amplification Kit (GE Healthcare, Piscataway, NJ, USA) was used for WGA. The lysis (400 mM KOH, 10 mM EDTA, 100 mM DTT), neutralization (0.4 ml of 1 M HCl and 0.6 ml of 1 M Tris·HCL, pH 7.5), and pushing buffers (1X Tris·EDTA, 0.2% Tween-20) were introduced to the dedicated inlets on the device. The amplification reaction was conducted on an AmpliSpeed slide cycler (Advalytix AG, Munich, Germany) for 2 h set at 34°C. After amplification, the samples were collected from the individual amplification units and transferred to a PCR plate followed by an inactivation step at 65°C for 10 min. For re-amplification of the on-chip amplified samples, 17 µl of the WGA mixture was added to 1 µl of on-chip amplified sample based on the manufacturer’s protocol and the reaction was carried out at 30°C on a Bio-Rad CFX 384 Real-Time System (Bio-Rad, Hercules, CA, USA,) for 70 min. This was followed by an inactivation step at 65°C for 10 min.

### Quantitation and Qualification

Qubit 2.0 Fluorometer and Qubit dsDNA HS Assay Kit (Invitrogen, Breda, NL) were used to quantify dsDNA. Real-time SYBR green qPCR was used for quantifying target-specific DNA. For *E.coli* WGA, primers against a small subunit (SSU), 369 bp, of the 16 S rRNA gene were used at a concentration of 500 nM. For single cancer cell amplification, primers designed for targeting small subunit of GAPDH (12p3), ERBB2 (17p12), CCND1 (11q13), MyC (8q24.21), PRMT2 (21q22.3), URB2 (1q42.13), FGFR1 (8p12), P53 (17p13.1), TRAM1 (8q13.3), and PAK1 (11q13-q14) were used at a concentration of 500 nM.

### Cell Culture and Preparation

The breast cancer cell line, SKBR-3(ATCCHTB-30) and MCF-7(ATCCHTB-22) cells, were cultured in Dulbecco’s Modified Eagle Medium (Sigma Aldrich, Zwijndrecht, NL), supplemented with 10% Fetal Bovine Serum (Sigma Aldrich), 2 mM L-Glutamin (Sigma Aldrich), 1% Penicillin-Streptomycin (Sigma Aldrich) at 37°C in 5% CO2 atmosphere. Before experimentation, cells were stained with CellTracker Orange CMTMR (Molecular Probes, Breda, NL) at 37°C for 30 min and detached by 0.05% of Trypsin/EDTA (Gibco, Paisly, UK). Thereafter, cells were washed once with the culture medium and re-suspended in PBS solution.

### Fluorescence In Situ Hybridization (FISH)

SKBR-3 and MCF-7 cells were cultured and harvested as described above. Both cell lines were fixed in suspension using Carnoy’s fixative (Methanol (Fisher Scientific, Loughborough, UK): Acetic Acid (Merck, Hohenbrunn, Germany), 3∶1) and dropped on a plain microscope slide. Slides were allowed to dry for 30 min before being incubated for 15 min in 2x SSC (20x SSC = 3 M sodium chloride (Merck), 0.3 M sodium citrate (Merck), pH 7.0), 0.5% Igepal (Sigma Aldrich) after which the slides were dehydrated in graded ethanol (70%, 85%, 100%) for one min each. 3.5 µl Her2/neu (ERBB2) (Kreatech, Amsterdam, NL) probe mix was applied to the slides followed by a Ø12 mm coverslip (Thermo Scientific, Breda, NL). After the edge of the coverslip was sealed with rubber cement (Kreatech), probes and target were denatured for 10 min at 76°C and allowed to hybridize for 16 h at 37°C. After hybridization the cover slips were removed and a stringent wash was applied using 0.4x SSC, 0.3% igepal at 72°C for 2 min. Slides were rinsed for 5 min in 2x SSC 0.1% igepal and dehydrated in graded Ethanol (70%, 85%, 100%) for 1 min each. Slides were finally mounted in DAPI/Antifade (Kreatech).

## Results and Discussion

### Device Characterization

**Figure 1:**
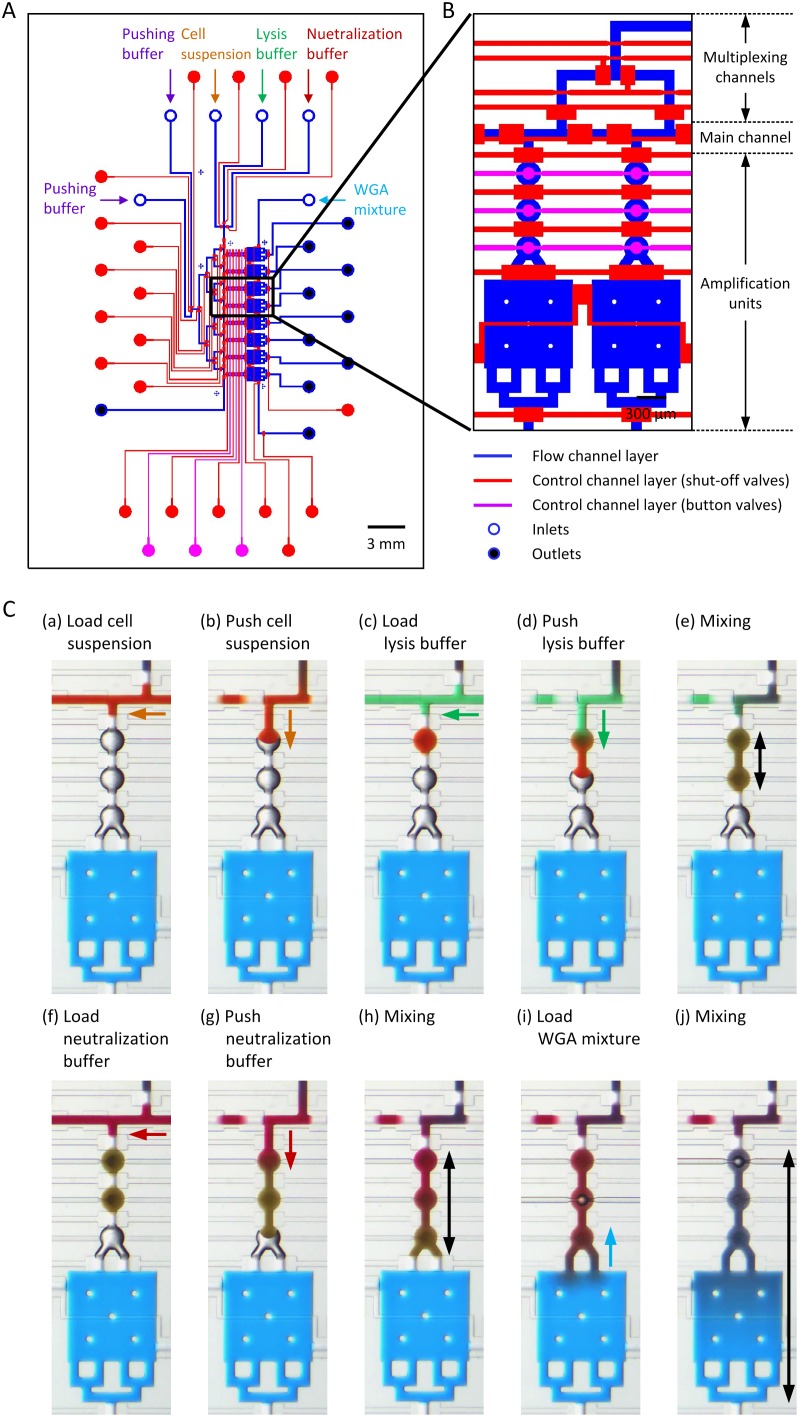
Design of single cell WGA device. (A) Schematic view of the device. The device contains 8 amplification units with 6 inlets and 10 outlets. (B) Magnified view of two amplification units. The blue color represent the flow channels, the red color represent the control layers for the shut-off valves and the pink color represent the control layers for the button valves. The amplification units consist of three circular chambers (1.2 nl) for cell suspension, lysis buffer, and neutralization buffer and one square chamber (20.25 nl) designed for WGA mixture. Mixing valves are located at the center of three circular chambers. (C) Device operation process. Bright-field images of an amplification unit with color dyes demonstrate operation process. (a–j).

**Figure 2:**
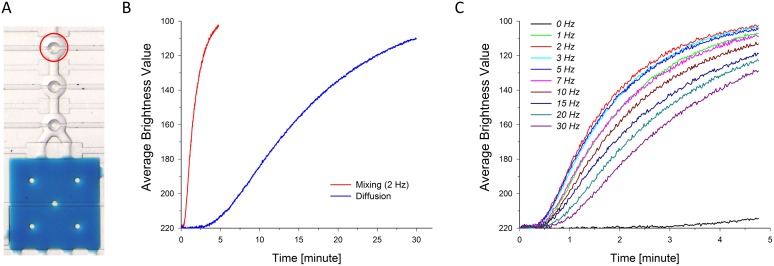
Button valve assisted mixing efficiency test. (A) Bright-field image of an amplification unit where the square chamber is filled with blue color dye and three circular chambers with water, taken at t = 0. Average brightness value of the first chamber (red circle) was monitored as a function of time after opening the valve. (B) Mixing efficiency of button valves. Measured average brightness values in the first circular chamber with mixing valve operation- indicated as black and without mixing valve operation indicated as green. (C) Mixing efficiency test at various frequencies of mixing valve operation. Average brightness values of the first chamber measured during 5 min.

The design and working principles of the parallel single cell WGA device are illustrated in Figure 1. The device includes 8 amplification units with 6 inlets (open blue circles) and 10 outlets (solid blue circles) (Figure 1A), and it consists of two layers, a fluidic layer (in blue) and a control layer (in red and pink). Each amplification unit (see Figure 1B) consists of three circular chambers (1.2 nl) and one square chamber (20.25 nl). The shut-off valves (in red) are operated pneumatically and allow for both manipulation of the cells and loading of the solutions. The mixing valves (in pink) located at the center of the three circular chambers are operated in the same manner. The main channel has three inlets for the respective introduction of the cell suspension, the lysis buffer and the neutralization buffer, which are connected to multiplexing channels to push the content to the 8 individual amplification units on the opposite side. The WGA products can be collected at the 8 individual outlet reservoirs. The device operation process is illustrated in Figure 1C, where color dyes were introduced in the device for visualization purposes, and imaged under a microscope. The blue dye represents the WGA mixture, the orange dye the cell suspension, the green dye the lysis buffer, the red dye the neutralization buffer and the purple dye the pushing buffer. First the blue dye was used to fill all rectangular chambers. Next, the orange dye was loaded in the main channel and pushed in the eight first circular chambers. The same operation was performed for the green and the red dyes. Finally, the solution present in the three circular chambers and the blue dye in the square chamber were mixed. Reagents were mixed achieved upon actuation of the three button valves, and this operation procedure is shown in a movie (Movie S1). For scWGA, we used the button valve actuation only for the WGA reaction step in Figure 1 (C–j). Because of the low Reynolds number found in the microfluidic channels, mixing occurs upon passive diffusion. To enhance the mixing efficiency for DNA amplification, button-shape valves were integrated in the closed amplification units of the device, at the center of the three circular chambers. Pressurizing the three button valves sequentially helps mixing reagents from the circular and square chambers. The mixing efficiency of the button valves was first assessed in mixing tests using a blue color dye. After the square WGA chamber was filled with blue color dye, the three circular chambers were filled with MiliQ water and the shut-off valve was opened between the circular and square chambers. Then, button valves were opened and closed sequentially at a given frequency. By observing the mixing of the colors the sequence of opening and closing button valves was optimized. To access mixing efficiency, the brightness value of the first circle chamber was recorded during mixing in the area indicated as a red circle line in Figure 2A. The time for complete mixing by diffusion solely was found to be approximately 30 min while button valve assisted mixing at 2 Hz was much faster and only took approximately 4 min, as shown in Figure 2B. Completion of mixing was set such that the brightness value measured using Image J software in the first chamber reached 110 (blue color) from the 220 measured from the transparent MilliQ. The operating valves were tested at frequencies between 0.1 and 30 Hz for 5 min and the mixing results are presented in Figure 2C. Mixing efficiency decreased below 0.5 Hz and above 5 Hz, and at least 4 min valve actuation was needed for optimal mixing. As the molecular weight influences the molecular diffusion coefficient and since the molecular weight of the food color dye is considerably smaller than that of the phi29 polymerase (M.W. 68,000), we also used Rhodamine conjugated dextran (M.W. 70,000) and Acridin Orange labeled DNA to assess the mixing in the device by fluorescence microscopy. Mixing was indeed slower compared to the food color dye and reached completion in approximately 20 min at 1 Hz. (Figure S1).

### Whole Genome Amplification on a Chip

**Figure 3:**
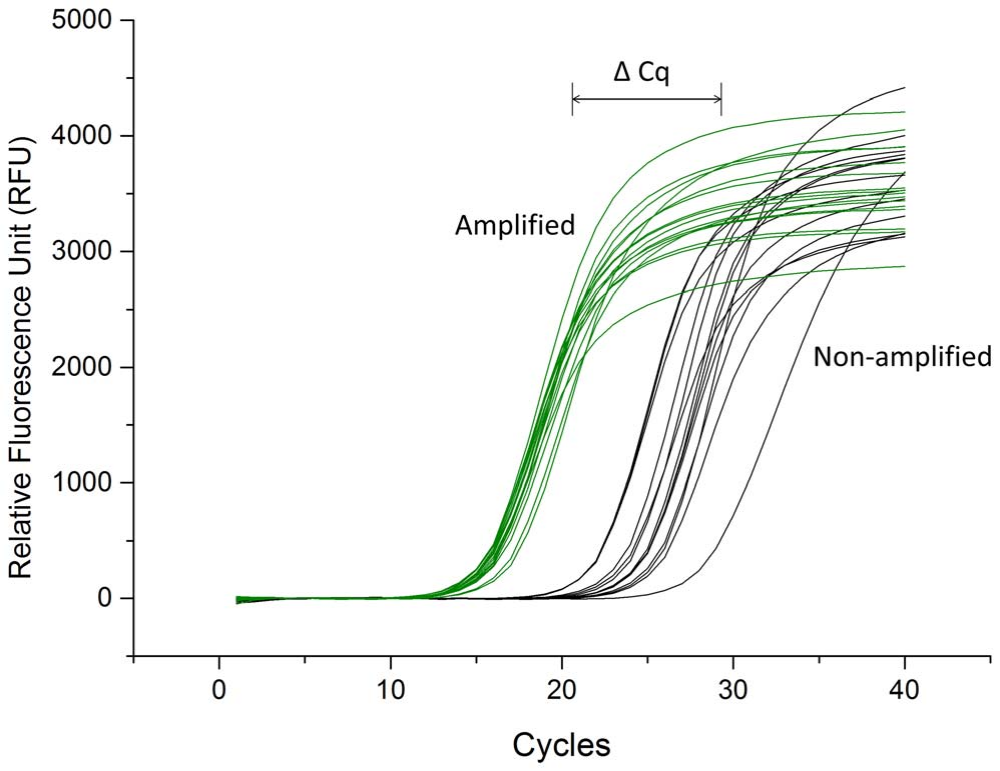
*E. coli* whole genome amplification on a chip. Real-time qPCR curves targeting SSU 16 S rRNA gene of individual collected samples from the device. On-chip amplified samples are represented as green curves (n = 16) while control samples without amplification are represented as black curves (n = 14).

Adaptation of the WGA protocol for isothermal DNA amplification with the phi29 enzyme onto a microfluidic device was performed using *E.coli* whole genome as a model sample. First, an *E.coli* DNA solution (6 ng/µl) was loaded into the main channel, and by closing the valves on the main channel an exact volume of 1.2 nl could be metered and loaded in the first chambers of the amplification units of the device, corresponding to the amount of DNA (7.2 pg) found in a single cancer cell. A buffer solution (1X Tris·EDTA, 0.2% Tween 20) was pushed through the first chamber of each amplification unit to fill the three circular chambers, while taking care the valves between the circular and square chambers were properly closed to isolate the WGA mix chambers. The WGA mix was subsequently introduced from the direct inlet to all square chambers before the reaction. Side valves of square chambers separated the individual amplification units. After opening the isolating valves between the circular and square chamber, the amplification reaction mixture consisting of phi29 polymerase, random hexamers and dNTPs in the square chamber and the *E.coli* DNA in the circular chambers started to diffuse and were actively mixed with the help of the button valves actuated at 1 Hz. After 2 h, the samples were pushed into a gel loading pipette tip placed at the outlet of the channel by inserting 5 µl of pushing buffer from the inlet, and the content of the pipette tip was subsequently transported to a PCR plate. After inactivation of the collected samples at 65°C for 10 min, qPCR targeting the SSU of 16 S rRNA gene was carried out to check the quality of the amplified product. The real-time qPCR curves using sixteen samples of 1 µl of on-chip amplified DNA are presented in Figure 3 (green lines), and they correspond to an average Cq of 14.77±0.55 (n = 16). For comparison, fourteen samples of 6 ng/µl of DNA solution in the 1.2 nl chamber were analyzed without any amplification. Those are represented by the black lines in Figure 3, and resulted in a average Cq value of 23.43±0.02 (n = 14). Larger amount of starting DNA show later Cq value on RT-qPCR and difference of Cq values (ΔCq) can quantify the difference of the amount between samples based on the standard curves. ΔCq of samples without amplification and samples after amplification were approximately 9. Therefore, the qPCR targeting SSU of 16 S rRNA gene resulted in a target-specific DNA amplification of 512 fold (2^ΔCq^ = 2^9^, 100% efficiency). Similar experiments performed without mixing using the button valves resulted in an average Cq value of 20.18±2.07 (n = 7), which clearly demonstrates the advantage of active mixing during the amplification step (Figure S2).

### WGA of Tumor Cells

**Figure 4:**
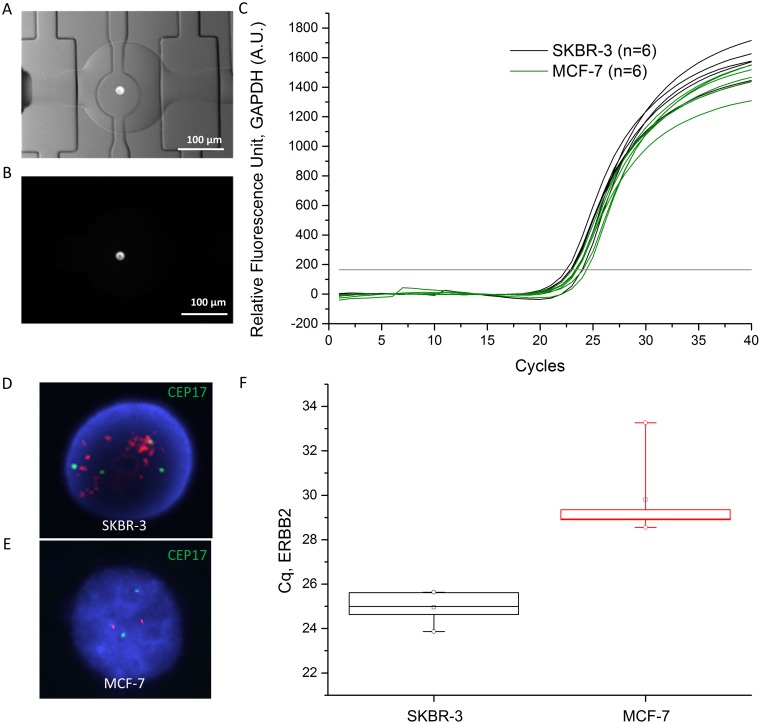
On-chip single cell whole genome amplification. (A) bright-field microscope image of a single SKBR-3 cell isolated in a microfluidic chamber, merged with a fluorescence picture. (B) fluorescence image of a single SKBR-3 cell stained with CellTracker Orange in the microfluidic chamber. (C and F) qPCR targeting GAPDH and ERBB2 genes of on-chip amplified DNA for quantification and characterization using 1 µl of on-chip amplified samples (5 µl) from individual SKBR-3 (n = 6) and MCF-7 (n = 6) as templates. (C) Real-time qPCR curves of GAPDH gene amplification. Amplified DNA of single SKBR-3 is represented using black curves while that of single MCF-7 cells is represented as green curves. Threshold line is in grey. (D and E) Images of fluorescence in situ hybridization (FISH) with ERBB2 probes (red) and centromere of chromosome 17 probes (green) for individual SKBR-3 (D) and MCF-7 (E). Nuclei were stained with Hoechst (blue) (F) Cq values of ERBB2 gene in a box plot. Cq values of ERBB2 were represented as black box in SKBR-3 (n = 6) and red box in MCF-7 (n = 5, * Cq of 1 sample is missing because of PCR error). (25%–75%: □, Median value:–, Mean value:□, Min/Max:°, Range of Min/Max:I).

To demonstrate the feasibility of the genetic characterization of individual tumor cells in the microfluidic device we used cells from the breast cancer cell line SKBR-3 and MCF-7. As the amount of DNA (6–7 pg) contained within a single cell is too low for a direct examination of its composition, the whole genome was amplified as described earlier. In the main channel the morphology and the immunophenotype of the cells can be examined and after verification that the cells fits our selection criteria individual cells can be moved into the first chamber of the amplification units. A cell suspension of SKBR-3 and MCF-7 cells stained with CellTracker Orange was injected into the main channel, and we identified cells to be subjected for further analysis by their fluorescence staining and morphology such as size and shape. After loading of the individual cells into the first chamber of an amplification unit, air was pushed to empty the main channel. As multiplexed flow lines are connected to the main channel each line can be opened individually by operating combination of the valves and only the cells of interest can be further processed. [32] Pictures of a SKBR-3 cell in an amplification chamber are shown in Figures 4A & B. After the cell was moved into the amplification unit another, the lysis buffer was loaded, as shown in Figure 1C, metered by closing the valves, and pushed using 1X Tris·EDTA, 0.2% Tween-20 to mix it with the cell. To accommodate the volume the valve between the first and second circle chambers was opened and lysis took place in the first two chambers for 10 min by diffusion. In the same way, the neutralization buffer was introduced and neutralization took place in the three circle chambers, also for 10 min based on diffusion. Valve actuation for mixing was not necessary for lysis and neutralization as diffusion time between 2 or 3 circular chambers was fast enough to perform the reaction. Actual lysis of the cells was monitored by microscopy. The WGA mixture was loaded into the square chambers by using separate inlets and outlets, and thereafter, the button valves were operated at 1 Hz during 2 h reaction to mix the cell lysate and the WGA mixture, and amplify DNA. Approximately 8.27 ng of DNA was obtained from the on-chip amplification, and, as a single cell contains 6–7 pg of DNA, more than 1000-fold amplification was achieved. Validation of template-specific DNA was performed by qPCR targeting the GAPDH gene locus as alterations of GAPDH have not been reported in both cell lines. The breast cancer cell line SKBR-3 and MCF-7 were used to demonstrate differences in the ERBB2 gene amplification. Figure 4C shows the real-time qPCR curves of GAPDH for six individual SKBR-3 cells (black curves) and six individual MCF-7 cells (green curves), analyzed using two different devices. Cq values for the GAPDH amplification quantified based on standard curves were 23.22±0.6. This implied that 33% of the total amount of DNA, measured by the Qubit assay, was template-dependent product. In Figures 4D and E FISH images after hybridization of ERBB2 probes (red) and centromere of chromosome 17 (green) of a SKBR-3 and MCF-7 cell are shown. Whereas 2 copies of chromosome 17 and 2 copies of the ERBB2 gene were observed in the MCF-7 cell, four copies of chromosome 17 and >10 copies of the ERBB2 gene were found in the SKBR-3 cell. Next, the same analysis was carried out using scWGA in our microfluidic device using ERBB2 specific qPCR, for individual SKBR-3 and MCF-7 cells. As shown in Figure 4 F, the averaged ERBB2 Cq value in the amplified DNA of individual SKBR-3 cells was 24.95±0.68 (n = 6) against 29.80±1.96 (n = 5) for individual MCF7 cells while the GAPDH Cq value for both cell lines was 23.22±0.6 (n = 12) shown in Figure 4C. The lower Cq value measured for SKBR-3 cells correlates well with the multiple copies of the ERBB2 gene observed by FISH (Figure 4D), which suggests that our on-chip WGA followed by qPCR for specific genes can be used to determine whether or not a gene is amplified.

**Figure 5:**
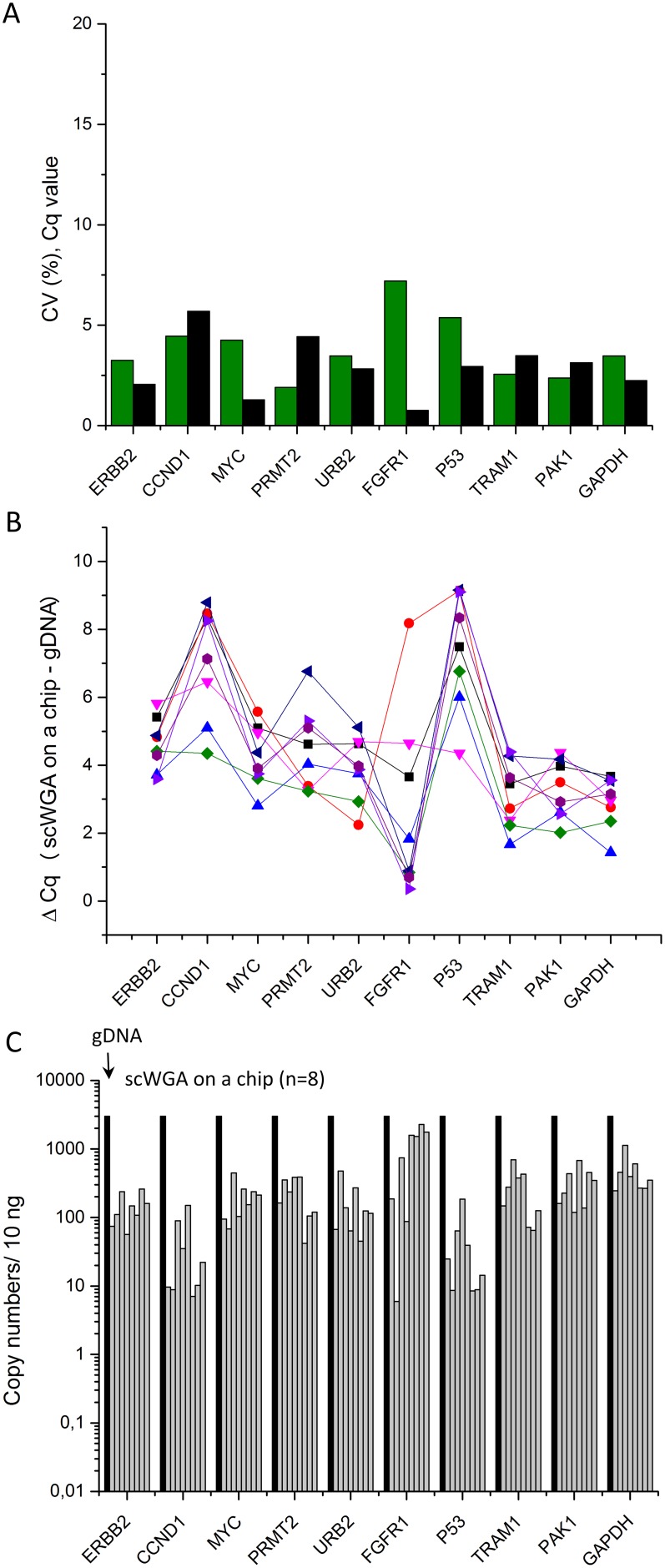
Representation of 10 loci in scWGA on a chip. Cq values of 10 ng DNA templates are determined by SYBR green qPCR targeting 10 genes. (A) The coefficient of variance (%) of Cq values of 4 samples amplified on the same chip. Samples on 2 different devices are indicated as green and black bars. (B) Individual line represents one amplified sample on a chip from a single cell. Y axis represents delta Cq values between 10 ng DNA amplified on a chip from single cell and genomic DNA. (C) Estimated copy numbers of individual samples are represented as gray bars. (n = 8) Black bars represent estimated copy numbers of genomic DNA in 10 ng.

For genetic analysis such as sequencing or high-throughput array analysis, larger amount of DNA (micrograms) are required. To evaluate suitability of re-amplified products for gene representation, we amplified off-chip 1 µl of 5 µl on-chip amplified DNA from a single SKBR-3 cell in 17 µl of WGA mix, followed by qPCR targeting 10 different loci on the whole genome. For this we choose 10 genes that are of interest for the cancer research field and located on different chromosomes. The amplified products of these genes can provide information on the representation of the starting DNA in the amplified products. 10 ng of 8 re-amplified samples collected from two devices (4 samples per device) were compared with 10 ng of genomic DNA (gDNA) of SKBR-3. Figure 5A shows the coefficient of variance (CV, %) of the Cq values on 4 samples in each device presented as green and black bars for the 10 loci considered. The average of the CV for those 10 genes were 3.8% for the first device (green) and 2.9% for the second device (black), and less than 7% variation was observed in any10 genes between the samples in the same device. The ΔCq values for the individual 8 samples compared to gDNA were determined for the 10 genes (see Figure 5B). The individual lines in Figure 5B correspond to one reaction on chip. ΔCq varied between genes but all 10 genes were amplified in 10 ng of scWGA samples (n = 8). The copy numbers of the target genes were estimated based on the qPCR standard curves of gDNA. The gray bars in Figure 5C represent the copy numbers of individual scWGA in 10 ng DNA while the black bars represent the copy numbers of genomic DNA in 10 ng. The averaged copy numbers for the 10 genes in 10 ng of DNA are: 128 copies of ERBB2, 47 copies of CCND1, 140 copies of MYC, 201 copies of PRMT2, 213 copies of URB2, 1182 copies of FGFR1, 42 copies of P53, 348 copies of TRAM1, 332 copies of PAK1, and 513 copies of GAPDH, which corresponds to percentages of representation of 4.8%, 1.4%, 6.6%, 7.5%, 5.4%, 34.1%, 1.5%, 9.1%, 10.7%, and 15.5%, respectively. Delayed Cq values and background amplification resulted in fewer copy numbers of genes compared to gDNA in 10 ng. Since the same result was observed when small copies of gDNA were amplified, we assume this bias is an artifact caused by the commercial kit. In addition, fluorescence-based DNA quantification, re-amplification, qPCR procedures, and quantification based on standard curves of genomic DNA might vary the results. Other non-isothermal and isothermal DNA amplification methods can be implemented in our microfluidic device though the reaction volumes and the reaction steps will need to be adapted and for non-isothermal temperature control will need to be introduced.

## Conclusions

Here, we presented a microfluidic device for the parallel processing of single cells to obtain sufficient amounts of DNA by whole genome amplification for downstream analysis. Button-shaped membranes were implemented as mixing valves, and sequential actuation of those valves enhanced the mixing efficiency. WGA in a 23.85 nl reaction volume was optimized by the use of *E. coli* gDNA. Individual breast cancer cells were captured, lysed and the DNA successfully amplified more than 1000-fold. 33% of the amplified DNA was template-specific, and genes relevant for drug response were successfully detected by qPCR after on-chip scWGA. DNA from the device can either be used to investigate the amplification of a set of specific genes or used for re-amplification suitable for whole genome sequencing. Reagent costs are significantly reduced by the 1000 times smaller reagent volume used in on-chip amplification and the ability to only select those cells that provide sufficiently high quality DNA for further investigation. For example, low quality DNA may not be able to amplify genes of interest for further analysis and experimental errors can occur during pre-amplification step. Automated manipulation, immunophenotyping, and parallel reaction benefit single cell whole genome amplification. Our microfluidic device could be improved by adding parallel reactors for future high-throughput genetic analysis of single cells.

## Supporting Information

Figure S1 Mixing efficiency test with fluorescence dye.
(DOCX)

Figure S2 Cq values of 16 S rDNA qPCR in a box plot.
(DOCX)

Movie S1 Device operation process.
(AVI)
