# Supplementary material for: Parallel Single Cancer Cell Whole Genome Amplification Using Button-Valve Assisted Mixing in Nanoliter Chambers
Source: PLoS One. 2014 Sep 18;9(9):e107958. doi: 10.1371/journal.pone.0107958 (PMC4169497; doi:10.1371/journal.pone.0107958)
Supplement: Figure S1 — Mixing efficiency test with fluorescence dye. (DOCX) [file pone.0107958.s001.docx]

**SUPPORTING INFORMATION – FIGURE S1**

**
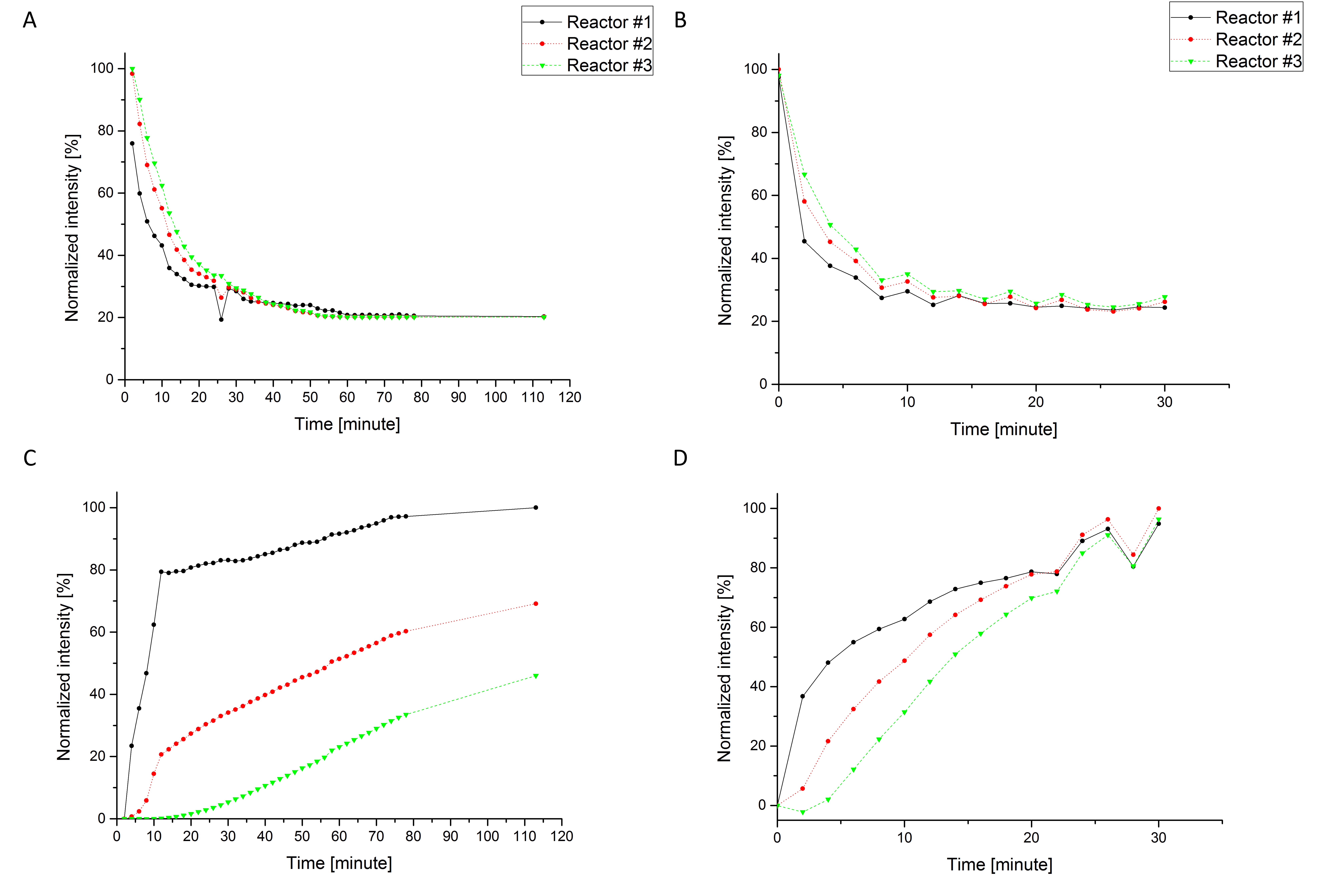
Figure S1.** Mixing efficiency test with fluorescence dye. Acridin Orange labled DNA were filled in 3 circle reactors and numbered as Reactor # 1,2, and 3 (from the closest to the farthest from square chamber). Rhodamine-conjugated dextran (M.W. 70,000, 1 mg/ml) was filled in a square chamber that is isolated with circle chambers by closing valve. The fluorescence intensity of DNA and Rhodamine-conjugated dextran in 3 circle reactors were measured under fluorescence microscope every 2 min. (T=0 is when the valve opens.) (A and C) Diffusion without mixing. (B and D) Button-valve mixing at 1 Hz. (A and B) Acridin Orange labeled DNA (10 mg/ml). (C and D) Rhodamine-conjugated dextran (M.W. 70,000).
