# Supplementary material for: Parallel Single Cancer Cell Whole Genome Amplification Using Button-Valve Assisted Mixing in Nanoliter Chambers
Source: PLoS One. 2014 Sep 18;9(9):e107958. doi: 10.1371/journal.pone.0107958 (PMC4169497; doi:10.1371/journal.pone.0107958)
Supplement: Figure S2 — Cq values of 16 S rDNA qPCR in a box plot. (DOCX) [file pone.0107958.s002.docx]

**SUPPORTING INFORMATION- FIGURE S2**

**
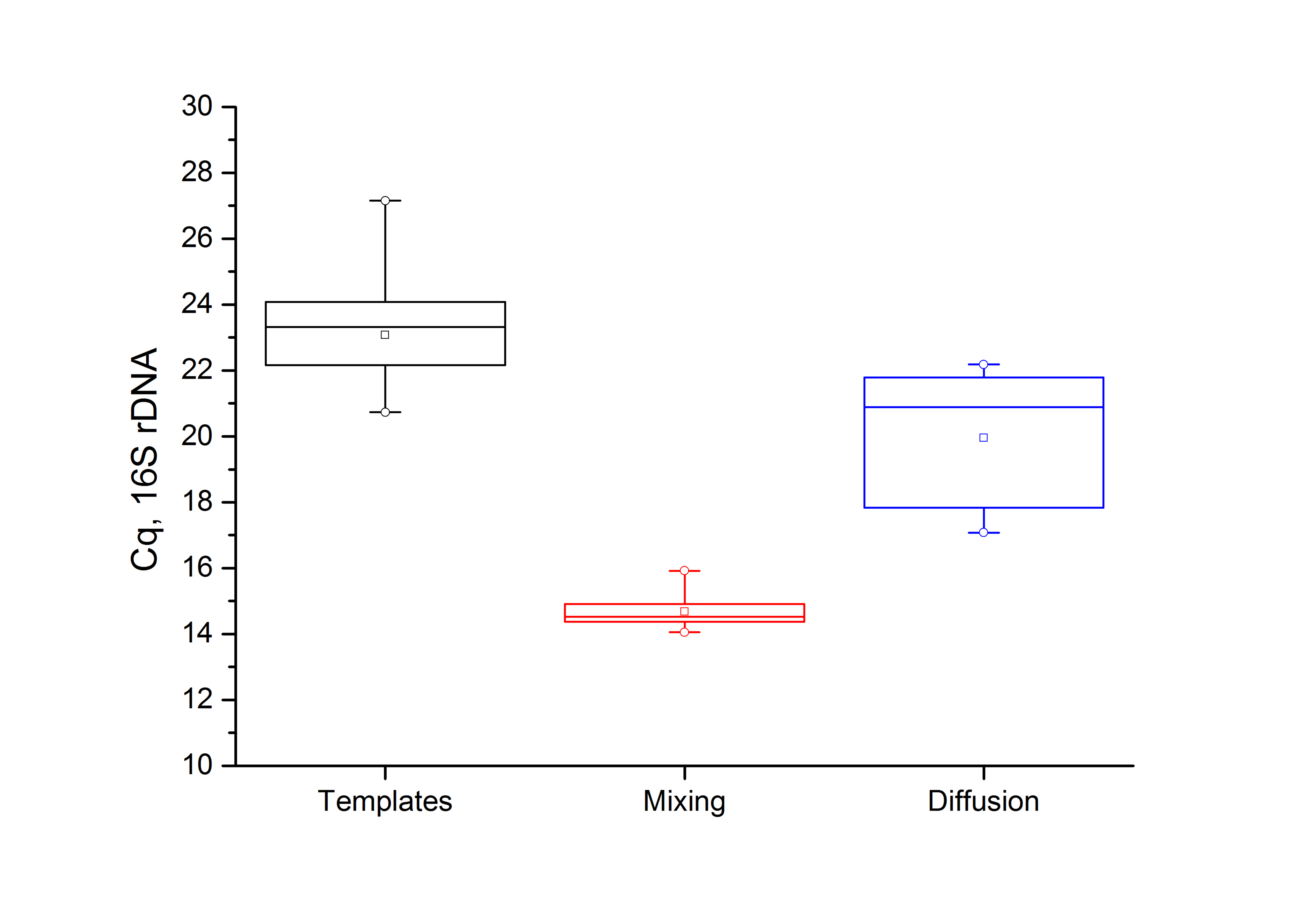
Figure S2.** Cq values of 16S rDNA qPCR in a box plot. (25 %-75 %: □, Median value:─, Mean value: ▫, Min/Max:◦, Range of Min/Max:I) According to the sample preparation methods, boxes were drawn as different colors. Collected starting *E.coli* DNA without amplification (templates) were presented as a black box (n=14). *E.coli* WGA with button-valve mixing were represented as a red box (n=16). *E.coli* WGA without button-valve actuation (diffusion) were represented as a blue box (n=14).
